# PET/CT Imaging of Activated Cancer-Associated Fibroblasts Predict Response to PD-1 Blockade in Gastric Cancer Patients

**DOI:** 10.3389/fonc.2021.802257

**Published:** 2022-01-26

**Authors:** Xiaoxiang Rong, Jinyu Lv, Yantan Liu, Zhaojun Wang, Dongqiang Zeng, Yuedan Li, Shaowei Li, Jianhua Wu, Zheyu Shen, Min Shi, Wangjun Liao, Zhenzhen Wu, Chunlin Wang

**Affiliations:** ^1^ Department of Oncology, Nanfang Hospital, Southern Medical University, Guangzhou, China; ^2^ School of Biomedical Engineering, Southern Medical University, Guangzhou, China

**Keywords:** gastric cancer, PD-1, immune checkpoint blockade, tumor microenvironment, biomarker, cancer-associated fibroblasts

## Abstract

**Background:**

Promising development in immune checkpoint blockade (ICB) therapy has shown remarkable results in the treatment of gastric cancer (GC). However, the objective response rate in GC remains unsatisfactory. Noninvasive imaging to predict responses to ICB therapy *via* tumor microenvironment (TME) assessment is needed. Accordingly, this study aimed to evaluate the role of ^68^Ga-FAPI-04 PET/CT in the assessment of the immunosuppressive TME in GC and to cross-correlate imaging findings with responses to ICB therapy.

**Methods:**

The correlation between fibroblast-activation-protein (FAP) expression and immunosuppressive cell infiltration was analyzed using The Cancer Genome Atlas (TCGA), Gene Expression Omnibus (GEO) database, and GC tissue microarrays. To characterize the TME, TMEscores were calculated based on RNA-seq data from four GC patients. A total of 21 patients with GC underwent ^68^Ga-FAPI-04 PET/CT before ICB treatment, and two of them were imaged after ICB therapy.

**Results:**

FAP expression was found to be closely correlated with poor prognosis and infiltration of immunosuppressive cells, including myeloid-derived suppressor cells (MDSCs), exhausted T cells, and regulatory T cells (Tregs) in GC. We also found a strong relationship (*R* ^2^ = 0.9678, *p* = 0.0162) between ^68^Ga-FAPI-04 uptake and TMEscore. Further analyses indicated that high ^68^Ga-FAPI-04 uptake was correlated with reduced therapeutic benefits from ICB therapy.

**Conclusions:**

^68^Ga-FAPI-04 PET/CT may be used to noninvasively image the cancer-associated fibroblasts immunosuppressive TME *in vivo* and also potentially serve as a predictive biomarker of survival and antitumor immune response among patients who received ICB therapies.

## Introduction

Metastatic gastric cancer (mGC) is ranked fifth in incidence and third in cancer-related mortality among malignancies worldwide (1). Despite the remarkable result development in immune checkpoint blockade (ICB) therapy has shown in certain cancers, the objective response rate (ORR) in mGC remains unsatisfactory (2). In the KEYNOTE-012 and KEYNOTE-059 clinical trials, pembrolizumab showed an ORR of 22% in PD-L1-positive mGC patients (3) and 11.6% in mGC patients irrespective of PD-L1 expression status with at least two lines of previous chemotherapy (4). Therefore, auxiliary markers to predict the response and prognosis of mGC patients with immunotherapy are urgently needed.

The tumor microenvironment (TME) plays an important role in the progression and therapeutic response of malignancies (5, 6). For example, clinical outcomes of patients with gastric and lung cancer vary with the changes in the numbers of CD8+ T cells, CD4+ T cells, macrophages, and cancer-associated fibroblasts (CAFs) infiltrating the TME (7–9). Our group originally designed a tool we named “TMEscore” to evaluate the comprehensive TME. The TMEscore was inferred to be a potent biomarker predicting the response to ICB and prognosis of GC patients, and patients with higher TMEscores had significantly better prognoses than those with lower TMEscores (9). However, the assessment of TMEscore requires repeated surgical biopsy/resection for RNA-seq, which is often not feasible or safe for mGC.

Over the last few years, noninvasive imaging techniques, such as PET imaging, have been proven to be sensitive tools to quantitatively monitor cell dynamics in the TME, for example, Rashidian et al. used PET to analyze the dynamics and distribution of CD8^+^T and CD11b^+^ cells in mouse models to predict the effect of immunotherapy (10–12). As a crucial member of the TME, CAFs contribute to tumorigenesis by producing growth factors, modeling the extracellular matrix, facilitating angiogenesis, and inhibiting antitumor immune responses (13). The presence of CAFs in the TME is usually a sign of unfavorable response to ICB therapy for the patients. As fibroblast activation protein (FAP) is highly specific to a large subset of CAFs, tracers targeting FAP that could potentially quantify CAFs could therefore provide a more comprehensive picture of the TME (12). In contrast to ^18^F-FDG, targeting FAP with the novel tracer, ^68^Ga-FAPI-04, has been shown to be suitable for marking CAFs and tumor imaging in preclinical GC models. Additionally, ^68^Ga-FAPI-04 is superior to standard PET imaging methods for the detection of primary intracranial tumors or metastatic tumors (14–17).

Increased FAP expression in GC has been associated with increased malignancy, poor prognosis, and tumor metastasis (18–20). In addition, FAP has been found to promote immunosuppression *via* myeloid-derived suppressor cell (MDSC) recruitment (21), Tregs, and tumor-associated macrophage (TAM) generation (22). Anti-FAP therapy, including nanoparticle-based photoimmunotherapy (23) and bispecific T-cell engagers (24), can significantly enhance T-cell infiltration and T-cell activation. Given the urgent need for noninvasive biomarkers of response to ICB and the central role of FAP + CAFs in this context, we performed PET with ^68^Ga-FAPI-04 to characterize the immunosuppressive TME in patients with mGC. In this study, we demonstrated that ^68^Ga-FAPI-04 PET/CT could be used to assess the immunosuppressive TME and monitor the responses to anti-PD-1 treatment in mGC patients.

## Methods

### Patients

We enrolled 21 patients with GC who received ICB therapy (FAPI cohort) from January 2020 through March 2021 at Nanfang Hospital. Contemporaneous ^68^Ga-FAPI-04 and ^18^F-FDG PET/CT were performed for all patients before immunotherapy, and two of them were also imaged after 2 months of immunotherapy, and the remaining patients underwent enhanced CT for efficacy evaluation. In addition, an RNA-seq-based TMEscore of four patients was conducted as described in previous studies (25). Tumor responses were evaluated according to RECIST 1.1 criteria. Seven patients were identified as having progressive disease (PD), and fourteen exhibited stable disease (SD) or partial remission after 2 months of immunotherapy. The data were analyzed retrospectively with approval from the Ethics Committee of the Nanfang Hospital. All data were gathered from patients with informed consent.

### Other Patient Cohorts Applied in This Study

Transcriptomic and corresponding clinical data of GC patients treated with immune checkpoint inhibitors in the NanoString cohort were used in this study. Gene expression data and clinical information from treatment-naive patients with stomach adenocarcinoma were downloaded from The Cancer Genome Atlas (namely TCGA-STAD cohort) (https://portal.gdc.cancer.gov/) and Asian Cancer Research Group (ACRG)/GSE62254 (GEO; https://www.ncbi.nlm.nih.gov/geo/). TMEscores of the two cohorts were conducted as previously described (25). The IMvigor210 dataset of urothelial cancer patients treated with an anti-PD-L1 agent (Atezolizumab) is available under the Creative Commons 3.0 license and can be downloaded from http://research-pub.gene.com/IMvigor210CoreBiologies.

### Estimation of TME Status

We quantify the TME status in GC patients using IOBR R package (26) (https://github.com/IOBR/IOBR), which offers multiple methodologies and signature construction tools, like (i) CIBERSORT (27), (ii) xCell (28), (iii) EPIC (29), (iv) MCPcounter (30), (v) pan-fibroblast TGF-β response signature (Pan-F-TBRs) (7), (vi) TMEscore (9), (vii) quanTIseq (31), etc.

### Radiopharmaceutical Synthesis and Quality Control

^18^F-FDG was automatically synthesized by the tracer synthesis module (Tracerlab FxFN, GE Healthcare, USA). DOTA-FAPI-04 was acquired from Nanchang Tanzhen Biological Co., Ltd. (Nanchang, China). Both chemical purities of ^18^F-FDG and ^68^Ga-FAPI-04 were greater than 95%. The radiosynthesis process of ^68^Ga-FAPI-04 has been described in detail in previously published articles (32–36). Quality control of the radiosynthesis was by radio-high-performance liquid chromatography (HPLC).

### PET/CT Imaging

All imaging was performed using a PET/CT scanner (uEXPLORER, United Imaging Healthcare, Shanghai, China). After low-dose CT (120 kVp, 80 mA) for attenuation correction, PET was accomplished through a 5-min single bed in 3-dimensional mode. All corrections are used for the reconstructed images and the anatomical location of the lesion. Using the list mode OSEM-PSF-TOF to reconstruct all data, patients were required to fast for at least 5 h, and their peripheral blood glucose levels were ensured to be normal for ^18^F-FDG PET/CT evaluation. The dose was evaluated depending on the patient’s weight (5.5 MBq/kg (150 μCi/kg) for ^18^F-FDG;1.8–2.2 MBq/kg (50–60 μCi/kg) for ^68^Ga-FAPI-04), and the PET scans were performed 1 h after injection. SUVmean and SUVmax were used to quantify the tumor tracer uptake. From 15 min before to 30 min after tracer application, 500-ml normal saline blended with 20 mg furosemide was infused. The patients were asked to self-report any unpleasant side effect 30 min after finishing the examination.

### Histology and Immunohistochemistry

In the FAPI cohort, the primary tumor lesions collected *via* gastroscopical biopsy were embedded with paraffin and sectioned at approximately 4 µm after being fixed in 4% paraformaldehyde. The slides and GC tissue microarrays (*n* = 31) were stained with rabbit anti-FAP (Abcam, ab53066; Cambridge, MA, USA), anti-CD11b (Abcam, ab133357), anti-CD33 (Abcam, ab269456), anti-CD163(Abcam, ab182422), and anti-PD-1 (Abcam, ab243644) primary antibodies overnight at 4°C and incubated with anti-rabbit secondary antibody for 1 h at room temperature. Finally, images were taken using a positive fluorescence microscope (Nikon Eclipse ci-L). We analyzed the intensity of FAP-positive cells in immunohistochemistry (IHC)-stained results using ImageJ software and calculated CD11b-, CD33-, CD163-, and PD-1-positive cells in five randomly selected high-power fields per section. The median value of FAP expression was defined as the cutoff value.

### Statistical Analysis

The expression of FAP was normalized using 10 housekeeping genes (ACTB, ABCF1, B2M, G6PD, GAPDH, GUSB, PGK1, RPLPO, TFRC, and TUBB). For data with normal distribution, the statistical significance of the two groups was calculated by unpaired Student’s *t*-tests. For data not displaying normal distribution, the statistical significance of the two groups was estimated using Wilcoxon rank-sum test. Correlation coefficients were computed using Pearson’s correlation analyses. The Survminer package (https://github.com/kassambara/survminer) was used to determine the cutoff values of each separate data based on the relevance between patient overall survival (OS) and FAP gene expression level. The pROC package (37) was applied to plot and visualize receiver operating characteristic (ROC) curves, evaluate the area under the curve (AUC), and measure the predictive value of OS of FAP, PD-1, PD-L1, TIM3, and PDCD1LG2 in the Nanostring cohort.

The survival information of the TCGA-STAD, ACRG, and IMvigor210 cohorts was collected to further analyze the relationship between the expression of FAP and overall survival rate. Kaplan-Meier curves were generated to compare the subgroups in all dataset, and the statistical significance of differences was presented using the logrank (Mantel-Cox) test. A univariate Cox proportional hazards regression model was used to estimate the hazard ratios for the univariate analyses.

All heat maps were developed with the pheatmap function (https://github.com/raivokolde/pheatmap). All statistical analyses were accomplished using R (https://www.r-project.org/) or SPSS software (version 25.0), and the *p*-values were two sided. Data were generated with two-sided *p*-value and regarded statistically significant at *p* < 0.05.

## Results

### High Expression of FAP Indicates Poor Prognosis

**Figure 1:**
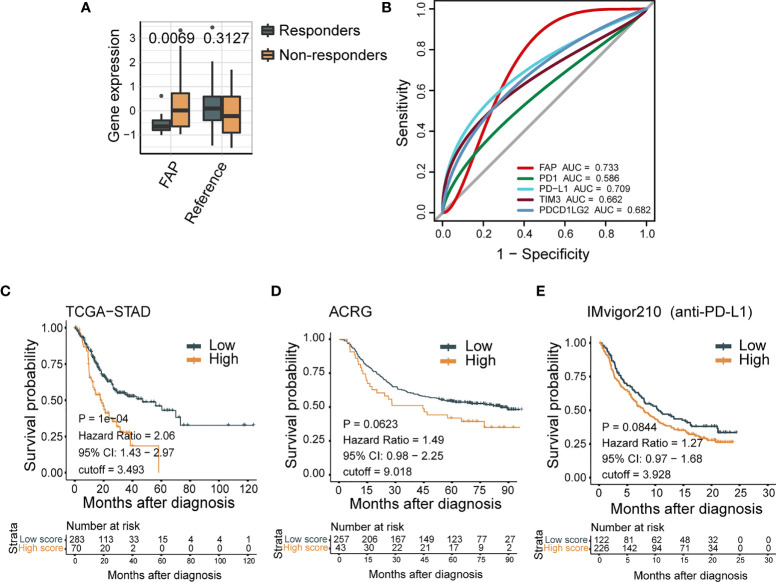
High expression of FAP indicates poor tumor prognosis. **(A)** FAP mRNA expression negatively correlates with response for ICB treatment in GC patients from Nanostring cohort (*N* = 48). **(B)** ROC curves measure the predictive value of FAP, PD-1, PD-L1, TIM3, and PDCD1LG2 in the Nanostring cohort (*N* = 48). The areas under the ROC curves are 0.733, 0.586, 0.709, 0.662, and 0.682 for the FAP, PD-1, PD-L1, TIM3, and PDCD1LG2, respectively. **(C–E)** FAP expression is negatively correlated with clinical outcome in a variety of tumors. In Kaplan-Meier analysis of the TCGA-STAD cohort **(C)**, ACRG cohort **(D),** and IMvigor210 cohort **(E)**, higher FAP expression indicates shorter overall survival.

Forty-eight patients with mGC in the Nanostring cohort (25) were categorized into responders (complete response/partial response) and nonresponders (SD/PD) according to their immunotherapy response. We then analyzed FAP mRNA levels in each group. FAP expression was significantly higher in GC patients who did not benefit from theICB therapy (*p* = 0.0069; **Figure 1A**). We next evaluated the prognostic value of FAP for immune-checkpoint therapy with ROC analysis in the NanoString cohort and observed a predictive advantage of FAP (AUC = 0.733) compared with PD-1, PD-L1, TIM3, and PDCD1LG2 (AUC = 0.586, 0.709, 0.662, and 0.682, respectively), which are widely accepted biomarkers for immunotherapeutic benefits (38–41) (**Figure 1B**). The analyses of the Nanostring cohort demonstrated that the mRNA expression of FAP could be a predictive biomarker for immunotherapeutic benefits, and after immune checkpoint therapy for GC, there were possible beneficial effects for patients with lower FAP mRNA levels.

We further confirmed the prognostic value of FAP for GC clinical outcomes by survival analysis. The values of FAP expressions are divided into three groups on average: high expression (top 1/3), middle expression (middle 1/3), and low expression (bottom 1/3). In the ACRG cohort, improved FAP expression demonstrated a decreased overall survival (HR, 2.06; 95% CI, 1.43–2.97; *p* < 0.001; **Figure 1C**). Similarly, in the TCGA-STAD cohort, high FAP expression showed a trend of poor overall survival (HR, 1.49; 95% CI, 0.98–2.25, *p* = 0.0623; **Figure 1D**). Moreover, a worse survival trend was observed in patients with higher FAP expression in the IMvigor210 cohort (HR,1.27; 95% CI, 0.97–1.68; **Figure 1E**), in which the patients exposed to urothelial cancer received anti-PD-L1 therapy. These results further confirmed that FAP expression could be a predictive biomarker for the clinical outcome of tumor patients.

### FAP Expression Is Closely Correlated With Infiltration of Immunosuppressive Cells in GC

In the TME, FAP expression is mainly restricted to activated fibroblasts, particularly CAFs (42–45). However, the relationship between FAP and the immune microenvironment requires further investigation.

**Figure 2:**
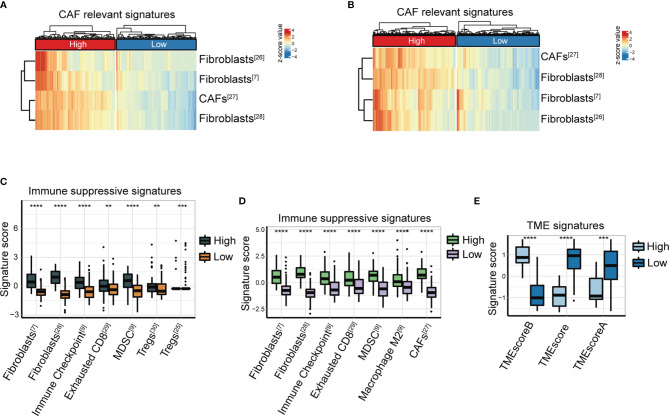
FAP expression is closely correlated with immunosuppressive signatures. **(A, B)** Heatmap of CAF-relevant signatures (7, 28–30) in the high or low FAP group from the ACRG cohort **(A)** and TCGA-STAD cohort **(B)**. **(C, D)** Immune suppressive signature score (7, 26–31) of two clusters distinguished according to FAP expression in the ACRG cohort **(C)** and TCGA-STAD cohort **(D). (E)** TME signature score between high and low FAP groups in the Nanostring cohort. ^**^ *p* < 0.01; ^***^ *p* < 0.001; ^****^ *p* < 0.0001.

We analyzed the features of FAP-associated immune infiltration in the TME in the TCGA-STAD and ACRG cohorts. The mRNA levels of FAP revealed a positive correlation with the cluster of CAFs in the TME, which was calculated using gene signatures as in previous studies (7, 28–30) (**Figures 2A, B**). We further explored the relationship between FAP and suppressive immune characteristics using immunosuppressive signature (7, 26–31). The results displayed that FAP expression was positively relevant to the abundance of immunosuppressive cells such as MDSCs (both *p* < 0.0001), exhausted T cells (*p* < 0.01 and *p* < 0.0001, respectively), as well as upregulation of immune checkpoints (both *p* < 0.0001) in both ACRG (**Figure 2C**) and TCGA-STAD (**Figure 2D**) cohorts. Moreover, FAP was positively correlated with the abundance of Tregs in the ACRG cohort (*p* < 0.01, **Figure 2C**) and infiltration of macrophages M2 in the TCGA-STAD GC cohort (*p* < 0.0001, **Figure 2D**).

In a previous study (9), we established the TMEscore, a methodology for the quantification of TME status, particularly for GC. The TMEscore has been demonstrated as a robust prognostic biomarker responsive to immune checkpoint inhibitors in GC (9). We investigated the association between FAP and TMEscore in the NanoString GC cohort. The data showed that patients with lower FAP expression had significantly higher TMEscore indicating active immune response and lower TMEscore indicating suppressive immune TME, compared with those with higher FAP expression (both *p* < 0.0001, **Figure 2E**).

**Figure 3:**
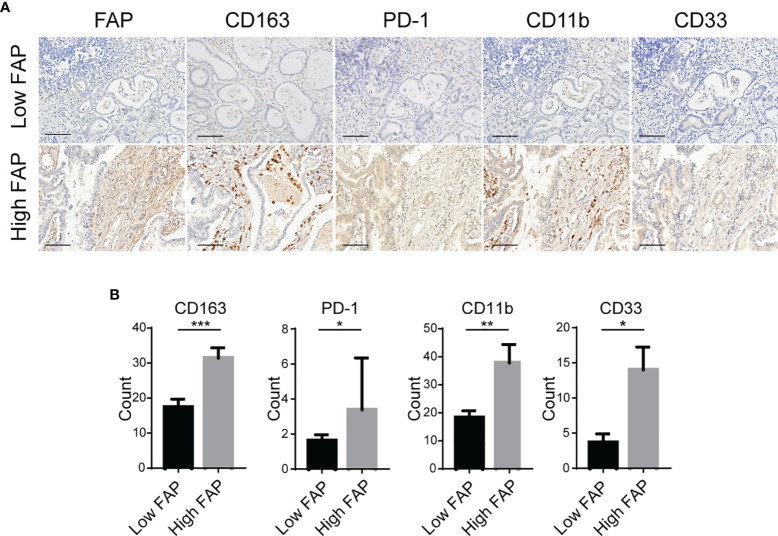
Association between the expression of FAP and immunosuppressive TME. **(A)** Representative images of IHC staining of FAP, macrophage M2 (CD163+), MDSC (CD11b+/CD33+) infiltration, and PD-1 expression in GC tissue microarrays. Scale bar = 100 µm. **(B)** Quantitative results of IHC for CD163, CD11b, CD33, and PD-1 in groups with low intensity and high intensity of FAP in GC tissue microarrays. ^*^ *p* < 0.05; ^**^ *p* < 0.01; ^***^ *p* < 0.001.

We further verified the relationship between FAP expression and tumor-infiltrating immune cells by IHC staining of GC tissue microarrays (*n* = 31). IHC staining of GC microarrays showed that the expression of FAP was considerably pertinent to infiltration of immunosuppressive cells, such as macrophage M2 (CD163+) and MDSCs (CD11b+/CD33+) (**Figures 3A, B**). In addition, the IHC intensity score of FAP was also higher in patients with upregulation of PD-1 expression in GC microarrays (**Figures 3A, B**). Overall, our data suggest that FAP is closely correlated with the immunosuppressive TME status in GC.

### ^68^Ga-FAPI-04 PET/CT Imaging Characterizes the Immunosuppressive TME in GC

The TME context of immunotherapy-naïve patients reflects therapy response (46). For example, changes in the number of Tregs (47), macrophage M2 (47), and CAF infiltration correlate with antitumor immune responses in GC (48). In previous studies, Ga-68 labeled fibroblast-activated protein inhibitor (^68^Ga-FAPI-04) with advantageous tumor-to-background contrast held great promise as a PET tracer due to the highly sensitive performance compared with ^18^F-FDG when detecting various types of cancer (36, 49, 50). To increase the translational value, we attempted to characterize the tumor environment using the noninvasive ^68^Ga-FAPI-04 PET/CT technique.

**Figure 4:**
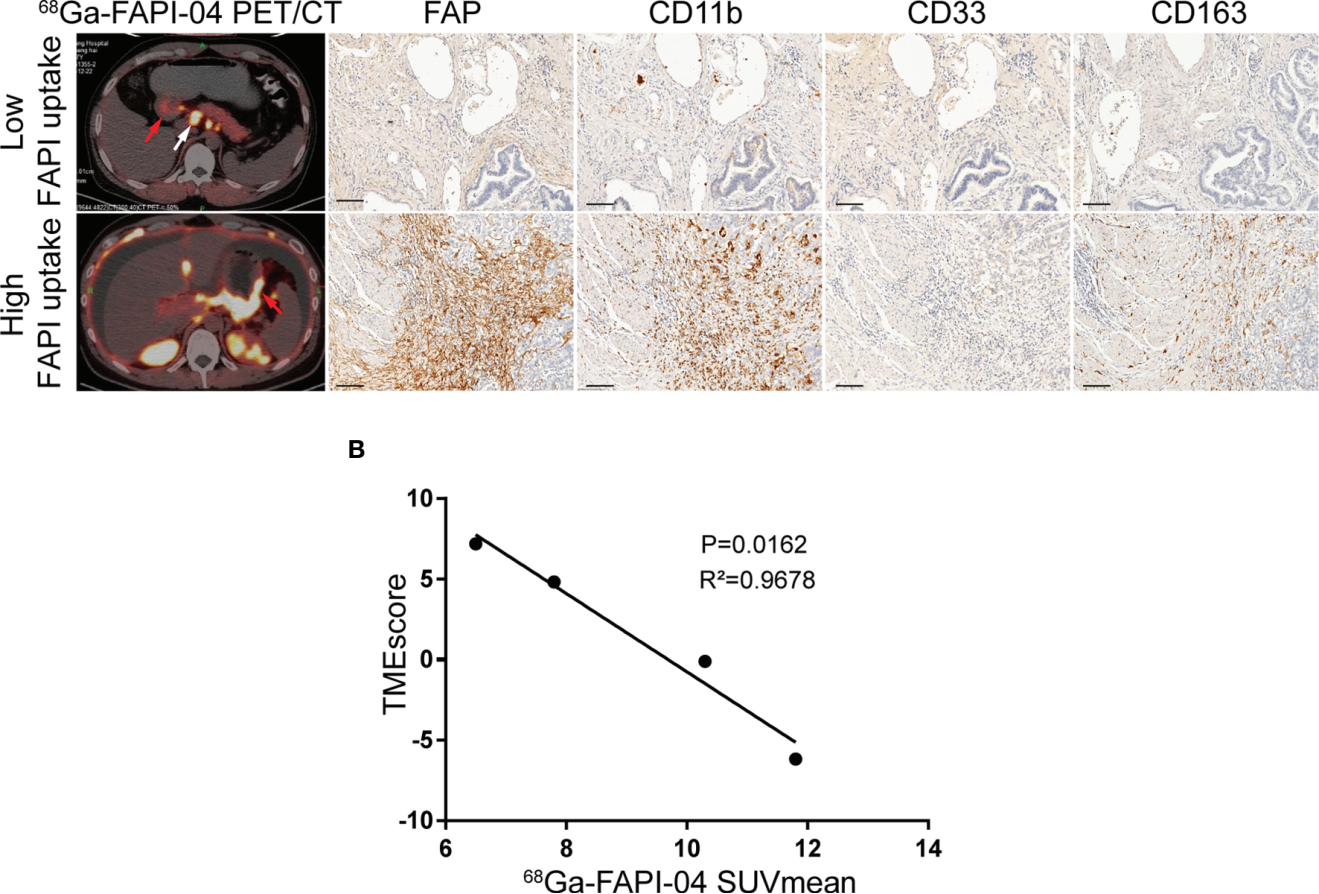
FAPI imaging characterizes the immunosuppressive TME in GC. **(A)** Representative ^68^Ga-FAPI-04 PET/CT images of patients with gastric cancer and IHC staining images for tumor specimens from gastroscopical biopsy of FAP, MDSC (CD11b+/CD33+), and macrophage M2 (CD163+) infiltration. Scale bar = 100 µm. The primary lesions are marked by red arrows, and the metastatic lesions are marked by white arrows. **(B)** Correlation analysis of TMEscore and the corresponding mean ^68^Ga-FAPI-04 uptake ratios in primary gastric cancer or liver metastases.

Here, we collected 21 patients (FAPI cohort) with mGC who undertook ^18^F-FDG and ^68^Ga-FAPI-04 PET/CT scans before immunotherapy. IHC staining of tumor specimens *via* gastroscopical biopsy was used to validate the infiltration of immunosuppressive cells in tumors and showed that the uptake of ^68^Ga-FAPI-04 positively correlated with FAP expression (**Figure 4A**). In addition, increased ^68^Ga-FAPI-04 uptake revealed higher levels of infiltration of MDSCs (CD11b+/CD33+), macrophage M2 (CD163+), and extensive PD-1 immunoreactivity in tumors (**Figure 4A**).

Hence, we speculated whether there were any correlations between the uptake of ^68^Ga-FAPI-04 and the TMEscore. By analyzing the data of tumor specimens from four patients in the FAPI cohort, TMEscore was found to be negatively correlated with the corresponding uptake of ^68^Ga-FAPI-04 before treatment (*R* ^2^ = 0.9678, *p* = 0.0162; **Figure 4B**). Therefore, from this cellular point of view, ^68^Ga-FAPI-04 PET/CT appears to be reliable as a means of assessing immunosuppressive TME in GC.

### FAPI Uptake Predicts Immunotherapy Response in GC

**Figure 5:**
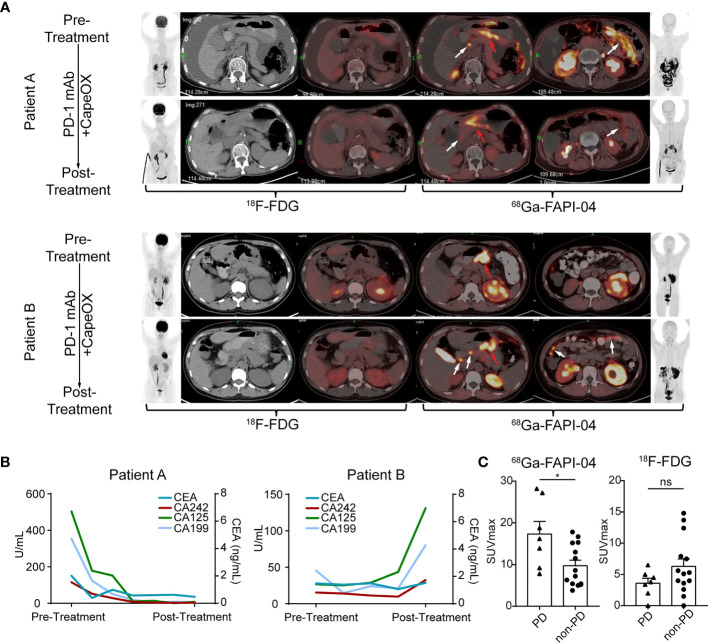
FAPI uptake predicts the immunotherapy response in GC. **(A)** (Patient A) Images of a 71-year-old female with gastric adenocarcinoma, that was sensitive to immunotherapy show low uptake of ^68^Ga-FAPI-04. The efficacy was confirmed by the reduced levels of ascites and the decreased uptake of ^68^Ga-FAPI-04 (red arrows, primary lesions; white arrows, metastatic lesions). (Patient B) Images of a 36-year-old female with gastric adenocarcinoma that was not sensitive to immunotherapy show high uptake of ^68^Ga-FAPI-04. The new metastatic lesions appear after immunotherapy (white arrows). Pretreatment and posttreatment images obtained at ^18^F-FDG and ^68^Ga-FAPI-04 PET/CT of GC patient. (Left: anterior maximum intensity projection image, axial unenhanced CT image, axial fused ^18^F-FDG image. Right: axial fused ^68^Ga-FAPI-04 PET/CT image, anterior maximum intensity projection image). **(B)** The curve of serum tumor markers of GC patients. Patient A who was sensitive to immunotherapy showed the decrease of serum tumor markers after treatment while patient B who was not sensitive to immunotherapy displayed increase of serum tumor marker. **(C)** SUVmax of ^68^Ga-FAPI-04 and ^18^F-FDG in gastric adenocarcinomas with different prognoses. Significant differences in SUVmax of ^68^Ga-FAPI-04 were detected between the PD and non-PD group (^*^ *p* < 0.05, ns, no significant).

To further confirm the value of ^68^Ga-FAPI-04 in predicting immunotherapeutic outcomes, the relationship between ^68^Ga-FAPI-04 uptake and immunotherapeutic efficacy was explored in the FAPI cohort. Two patients in our FAPI cohort undertook both ^18^F-FDG and ^68^Ga-FAPI-04 PET/CT imaging before and 2 months after receiving CapeOX plus anti-PD-1 therapy. Pretreatment ^18^F-FDG PET/CT showed a thickened gastric wall in both patients A and B (**Figure 5A**, patient A, pretreatment SUVmax = 5.6; patient B, pretreatment SUVmax = 3.4), and a medium amount of ascites in patient A (**Figure 5A**). In the pretreatment ^68^Ga-FAPI-04 PET/CT imaging, the thickened gastric wall displayed a notably high image contrast (**Figure 5A**), and ^68^Ga-FAPI-04 uptake of the primary lesion in patient B was higher than that in patient A (**Figure 5A**, patient A, pretreatment SUVmax = 11.5; patient B, pretreatment SUVmax = 16.2).

After 2-month treatment, tumor entities with weak pretreatment ^68^Ga-FAPI-04 uptake (patient A) were sensitive to ICB, and ^68^Ga-FAPI-04 PET/CT demonstrated local tumor control with a decline in tumor volume and uptake of peritoneal metastasis and ascites formation, and the tumor marker (CEA, CA242, CA125, and CA199) levels also decreased remarkably (**Figure 5B**). In contrast, for the tumor lesion with high pretreatment ^68^Ga-FAPI-04 uptake (patient B), the posttreatment ^68^Ga-FAPI-04 scan demonstrated newly developed peritoneal metastases (**Figure 5A**, white arrows), compared with the first scan performed 2 months earlier. Furthermore, tumor marker levels also greatly increased after treatment (**Figure 5B**).

We further investigated the association between ^68^Ga-FAPI-04 uptake and immunotherapy response in the FAPI cohort and found that immunotherapy-insensitive patients (PD) showed significantly higher pretreatment uptake of ^68^Ga-FAPI-04 compared with immunotherapy-sensitive patients (non-PD) (*p* = 0.0149, **Figure 5C**). By contrast, through comparison of pretreatment ^18^F-FDG SUVmax, a significant difference was absent between patients with distinct responses to ICB (*p* = 0.1554, **Figure 5C**). Altogether, our data strongly indicate that ^68^Ga-FAPI-04 PET/CT could serve as a predicting marker to clarify the efficacy of immunotherapy in GC. More specifically, patients with lower pretherapeutic ^68^Ga-FAPI-04 uptake have a better response to anti-PD-1 treatment. Thus, ^68^Ga-FAPI-04 PET/CT prior to therapy may represent a new noninvasive potential predictive candidate for anti-PD-1 treatment efficacy in GC.

## Discussion

To date, efficient imaging biomarkers to characterize the immunosuppressive TME of GC that can monitor the responses to anti-PD-1 treatment are lacking. In this study, we demonstrate the potential of ^68^Ga-FAPI-04 PET/CT in the noninvasive assessment of immunosuppressive TME in GC. Our findings indicate that high expression of FAP predicts poor prognosis in GC patients who undergo ICB therapy. Based on TCGA, GEO database analysis, and IHC staining of GC microarrays, we found that FAP expression is closely positively correlated with immunosuppressive cell infiltration. Interestingly, ^68^Ga-FAPI-04 PET/CT was found to be negatively correlated with TMEscore, suggesting a role for ^68^Ga-FAPI-04 PET/CT in the identification and stratification of patients in accordance with their individual immunosuppressive status.

The development of ICBs is a milestone in the field of immunology. ICB is known to trigger antitumor immunity by interfering with coinhibitory pathways and facilitating the elimination of cancer cells by the immune system. Despite the clinical success of ICB therapies, only certain patients benefit from ICBs due to the complex variation of TME. The TME can be categorized into three main types according to the infiltration of immune cells: immune inflammation, immune exclusion, and immune desert. Each type has a special mechanism to prevent the immune response from eradicating tumor cells (51). It is clearly understood that CAFs play an important role in the formation of an immunosuppressive TME. A sustained network is not only woven between cancerous and immune cells but also among stromal cells, such as CAFs and other types of immune cells. CAFs, for example, helped recruit monocytes to differentiate into M2-like macrophages, which could affect PD-1 pathways to perform immunosuppressive functions (52). As a marker of CAFs, FAP-positive CAFs contribute to immunosuppression by secreting CXCL12 in a pancreatic cancer model (53), enhancing recruitment of MDSCs *via* STAT3-CCL2 signaling (21) and promoting the generation of Tregs and TAMs (22). In line with our findings, FAP expression in mGC was found to be positively associated with infiltration of Tregs, macrophage M2, and MDSCs.

Assessment of the TME allows for the prediction of patients who can benefit from ICB therapies. Emerging evidence support the view that TME plays an important role in ICB therapies (9). Perhaps the most prevailing strategy to evaluate TME involves IHC detection for markers like CD8 and PD-L1, although this approach is admittedly limited (54). Biomarkers, for example, that can predict responsiveness to ICB in mGC patients include Epstein-Barr virus (EBV) infection and PD-L1 high expression, while single stain IHC could not cover all the patients who may benefit from ICB therapies. In previous research, TME infiltration of 1524 GC patients was broadly gauged and divided into three TME types with unique genomic and clinicopathologic features: the high TMEscore defined by immune activation and response to virus and IFN-γ was constructed, while the low TMEscore was characterized by stimulation of transforming growth factor β, epithelial–mesenchymal transition, and angiogenesis pathways (9). Nevertheless, the assessment of TMEscore requires surgical biopsy/resection for RNA-seq; however, it is difficult to realize continued progress in TME monitoring. Booming techniques brought far less invasive and more robust assessment of the TME, especially with advances in molecular imaging.

As a noninvasive and real-time monitoring method, molecular imaging tracks the entire process of immunological responses and evaluates TME to predict ICB outcomes. As the field develops, discreet selection of biomarkers is vital. Nucleoside analogs such as ^18^F-AraG, ^18^F-FLT, and ^18^F-AraC have been developed for immune cell imaging (55–57). Since the uptake of these tracers is determined by substantial nucleoside transporters and DNA salvage pathway enzymes, uptake in certain types of cancers with similar features may perplex the result of immune cells imaging. Furthermore, molecular imaging of the TME is arduous to apply in the clinic, considering the imaging effect and tracer safety. Imaging tracers directly show that the metabolism condition of certain cells may be more predictive.

Our data *in vivo* identified FAP as an encouraging biomarker for imaging activated CAFs, with ^68^Ga-FAPI-04 PET/CT appearing to be a feasible strategy for clinic use (58). Moreover, ^68^Ga-FAPI-04 PET/CT showed superior diagnostic efficacy to ^18^F-FDG PET/CT in various types of cancers, including GC (36). For the first time, we used ^68^Ga-FAPI-04 PET/CT as a noninvasive biomarker to predict outcomes of ICB therapy; patients with higher FAPI uptake might fail to undergo ICB therapy. In conclusion, we were able to noninvasively estimate the immunosuppressive TME using ^68^Ga-FAPI-04 PET/CT. Furthermore, ^68^Ga-FAPI-04 PET/CT not only helps us to quantify the CAFs in TMEs but also potentially serves as a predictive biomarker of survival and antitumor immune response among patients who received ICB therapies. Additional studies in patients with mGC are needed to verify the prediction efficiency observed in this study.

## Data Availability Statement

The datasets presented in this study can be found in online repositories. The names of the repository/repositories and accession number(s) can be found in the article/supplementary material.

## Ethics Statement

The studies involving human participants were reviewed and approved by the Ethics Committee of the Nanfang Hospital. The patients/participants provided their written informed consent to participate in this study.

## Author Contributions

CW, ZZW, and XR proposed this study. XR, JL, and YTL wrote the paper. ZJW, DZ, and YDL performed the experiments and analyzed the data. SL, JW, MS, ZS, and WL enrolled patients and provided the images of PET/CT. All authors contributed to the article and approved the submitted version.

## Funding

This work was supported by the National Natural Science Foundation of China (No. 82073375 to XR, No. 81702398 to CW), China Postdoctoral Science Foundation (2019M663001 to XR), and the Science and Technology Planning Project of Guangzhou (No. 202102020532).

## Conflict of Interest

The authors declare that the research was conducted in the absence of any commercial or financial relationships that could be construed as a potential conflict of interest.

The handling editor declared a shared parent affiliation with the authors at the time of review

## Publisher’s Note

All claims expressed in this article are solely those of the authors and do not necessarily represent those of their affiliated organizations, or those of the publisher, the editors and the reviewers. Any product that may be evaluated in this article, or claim that may be made by its manufacturer, is not guaranteed or endorsed by the publisher.
