# Corrigendum: PET/Ct Imaging of Activated Cancer-Associated Fibroblasts Predict Response to PD-1 Blockade in Gastric Cancer Patients

**DOI:** 10.3389/fonc.2022.895938

**Published:** 2022-04-07

**Authors:** Xiaoxiang Rong, Jinyu Lv, Yantan Liu, Zhaojun Wang, Dongqiang Zeng, Yuedan Li, Shaowei Li, Jianhua Wu, Zheyu Shen, Min Shi, Wangjun Liao, Zhenzhen Wu, Chunlin Wang

**Affiliations:** ^1^ Department of Oncology, Nanfang Hospital, Southern Medical University, Guangzhou, China; ^2^ School of Biomedical Engineering, Southern Medical University, Guangzhou, China

**Keywords:** gastric cancer, PD-1, immune checkpoint blockade, tumor microenvironment, biomarker, cancer-associated fibroblasts

There is an error in the Funding statement. The correct number for National Natural Science Foundation of China to Xiaoxiang Rong is No. 82073375.

The authors apologize for this error and state that this does not change the scientific conclusions of the article in any way. The original article has been updated.

## Funding

This work was supported by the National Natural Science Foundation of China (No. 82073375 to XR, No. 81702398 to CW), China Postdoctoral Science Foundation (2019M663001 to XR), and the Science and Technology Planning Project of Guangzhou (No. 202102020532).

## Publisher’s Note

All claims expressed in this article are solely those of the authors and do not necessarily represent those of their affiliated organizations, or those of the publisher, the editors and the reviewers. Any product that may be evaluated in this article, or claim that may be made by its manufacturer, is not guaranteed or endorsed by the publisher.

